# Complete plastome sequences of two *Psidium *species from the Galápagos Islands

**DOI:** 10.12688/f1000research.15653.1

**Published:** 2018-08-30

**Authors:** Bryan Reatini, Maria de Lourdes Torres, Hugo Valdebenito, Todd Vision

**Affiliations:** 1 Department of Biology, University of North Carolina at Chapel Hill, Chapel Hill, North Carolina, 27514, USA; 2 Universidad San Francisco de Quito, Quito, Ecuador

**Keywords:** plastome, Psidium, Galapagos, guayabillo

## Abstract

We report the complete plastome sequences of an endemic and an unidentified species from the genus *Psidium* in the Galápagos Islands ( *P. galapageium* and *Psidium sp.* respectively).

## Introduction

Over a quarter of all vascular plant species are endemic to islands, making them hotspots of plant diversity and conservation ( Kreft *et al.*, 2008). In the Galápagos Islands, there are roughly 560 native species of plants of which approximately 32% are endemic ( Lawesson *et al.*, 1987). However, many of these endemic species have remained relatively unstudied since they were originally given scientific descriptions, making the study of the evolutionary histories of these unique taxa difficult. In the present study, we constructed the complete plastome sequences of two species of *Psidium* (guava) from the Galápagos Islands, one endemic and one currently unidentified in hopes of facilitating future work on the evolutionary relationships of these species.

## Methods

This research is authorized under the permit: MAE-DNB-CM-2016-004 in compliance with Ecuadorian regulations.

Leaf samples were collected during May of 2017 from the Galápagos endemic *Psidium galapageium* Hook (commonly known as guayabillo) on the island of San Cristobal (0.89094°S, 89.43769°W) and from an unidentified *Psidium* species on the island of Santa Cruz (0.62313°S, 90.38581°W). Based on morphological similarity, the *Psidium sp.* individual is suspected to be *P. acidum* ( Landrum, 2016), but no reference or barcode sequence from *P. acidum* is available for confirmation.

Leaf tissue was desiccated immediately after harvesting using silica gel. DNA extractions were performed using a Qiagen DNeasy Plant mini kit (Qiagen, Inc.). Sequence data was generated in the form of paired-end, 150 bp reads using a KAPA library prep kit (Roche Sequencing) and sequenced on an Illumina HiSeq 4000 platform (Illumina, Inc.).

**Figure 1.**
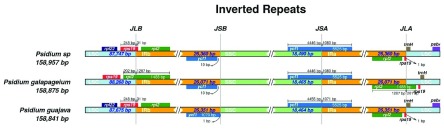
Genomic structure of junction sites between the long single copy (LSC, light blue), short single copy (SSC, light green), and inverted repeat (IBa and IRb, orange) regions. Proximate genes are shown. The circular genomes have been linearized for illustration.

Reads were quality and adapter trimmed using *Trim Galore!* version 0.4.3 with a minimum *phred* score value of 20 and minimum read length of 50 bp. Filtered reads were then aligned to the *Psidium guajava* plastome reference available at NCBI (Accession: KX364403) using the mem function within *BWA* version 0.7.15 ( Li & Durbin, 2009). Consensus plastome sequences were generated using the mpileup function within *samtools* version 1.8 followed by the call and consensus functions within *bcftools* with a minimum depth of coverage of 10x ( Li *et al*., 2009). Using *IRscope* ( Amiryousefi *et al.*, 2018), the *P. galapageium* and *Psidium sp.* plastomes respectively were confirmed to contain a large single copy of 88,268 bp and 87,747 bp and a small single copy of 18,465 bp and 18,490 bp separated by two inverted repeats of 26,071 bp and 26,360 bp for total lengths of 158,875 bp and 158,957 bp ( Figure 1).

Annotations were generated using the program *Plann* ( Huang & Cronk, 2015). Of the 132 gene features annotated previously in the *Psidium guajava* (guava) chloroplast genome on NCBI (Accession: KX364403), all were recovered in the *Psidium sp.* and *P. galapageium* plastome sequences. The non-identity of the two taxa sampled is evidenced by the absolute pairwise sequence divergence of the concatenated sequences of three conserved genes (MatK, psbA, and rbcL) which have been successfully used as barcodes previously in *Psidium* ( Kress *et al.*, 2009). Sequences were aligned using MUSCLE within MEGA version 7.0.26 ( Tamura *et al.*, 2007), and the number of nucleotide differences were counted between these alignments to estimate divergence. A total of 35 differences were observed among 4011 sites (0.87% uncorrected divergence) between *P. guajava* (Accession: KX364403) and *P. galapageium,* 45 differences (1.1%) between *P. guajava* and *Psidium sp.*, and 40 differences (0.99%) between *P. galapageium* and *Psidium sp.*

## Data availability

Voucher specimens for *P. galapageium* and *Psidium sp.* are available at the Charles Darwin Research Station herbarium (Index Herbariorum code CDS) with accession numbers 3053515 and 3053562, respectively. The corresponding plastome sequences for *P. galapageium* and *Psidium sp.* are available at NCBI with accession numbers MH491846 and MH491847 *,* respectively.
