# Supplementary material for: Complete plastome sequences of two Psidium species from the Galápagos Islands
Source: F1000Res. 2018 Aug 30;7:1361. [Version 1] doi: 10.12688/f1000research.15653.1 (PMC6173110; doi:10.12688/f1000research.15653.1)
Supplement: Peer review history

# Peer review: Referee response for version 1

**DOI:** 10.5256/f1000research.17078.r39032

**Published:** 2018-10-03

**Authors:** Carolyn Proença

**Affiliations:** 1 Department of Botany , Institute of Biological Sciences, University of Brasília (UnB), Brasília, Brazil

This appears to be a sound paper. Methods are modern, standard and referenced with versions cited when appropriate. Sequences are vouchered by herbarium specimens and have been deposited in Genbank. I have checked the accession numbers given and they are correct. Figure 1 is very clear and detailed, and the legend is adequate and complete.

 My most important comment is to point out that very recently, Landrum (2017, in Canotia 13:1-101) has treated *Psidium galapageium* Hook.f. as a synonym of widespread *P. oligospermum* DC. He comments in his paper that “As recognized here *Psidium oligospermum* is a widespread and variable species” and that “A geographically broad study with molecular techniques of *Psidium oligospermum*, including related species ... would be valuable.” Reatini’s paper relates directly to this comment by Landrum (2017) and the fact that the independent species status of *P. galapageium* is not accepted by all should be made. Other studies however (Proença *et al. *2014 Flora de Sergipe: Myrtaceae; and Tuler *et al.* 2017, Flora of Espírito Santo: *Psidium* (Myrtaceae). Rodriguesia 68:1791-1805) do not include *P. galapageium* within *P. oligospermum.*

 A minor quibble is with authorities for scientific names in the paper, that are either erroneous or missing. *Psdium galapageium* Hook.f., is the correct form (authority erroneously given in the paper as Hook who is actually a different botanist from Joseph Hooker who described the species). *Psidium acidum *(Mart. ex DC.) Landrum (authorities absent in the paper) is how this species should be cited.

I have read this submission. I believe that I have an appropriate level of expertise to confirm that it is of an acceptable scientific standard.

# Peer review: Referee response for version 1

**DOI:** 10.5256/f1000research.17078.r37720

**Published:** 2018-10-03

**Authors:** Michael O. Dillon

**Affiliations:** 1 Integrative Research Center, The Field Museum, Chicago, IL, USA

This paper reports the results of sequencing efforts of a species native to the Galapagos Islands.  The ability to distinguish new species and especially "crypto-species" that are not always obvious due to similar comparative morphologies.  The paper should be published and this is the appropriate venue.

I have read this submission. I believe that I have an appropriate level of expertise to confirm that it is of an acceptable scientific standard.
